# Group play as a buffer against stress in preschoolers: reductions in externalizing but not internalizing symptoms

**DOI:** 10.3389/fpsyg.2026.1874167

**Published:** 2026-06-25

**Authors:** Valeriya Plotnikova, Aleksandr Veraksa, Vera Sukhikh

**Affiliations:** Laboratory of Childhood Psychology and Digital Socialization, Federal Scientific Center of Psychological and Multidisciplinary Research, Moscow, Russia

**Keywords:** cultural-historical psychology, early childhood, externalizing symptoms, internalizing symptoms, perceived stress, pretend play, role play

## Abstract

This study examined the potential of group-based role play in kindergarten settings as a means of reducing stress and its manifestations in preschool children. The study was grounded in the cultural-historical approach, which considers role play a leading activity in preschool age and a key context for the development of self-regulation and socio-emotional competence. At the initial stage, 210 preschool children were assessed using the Perceived Stress Scale for Children (PSS-C). Children with high perceived stress scores were selected for the intervention. The final sample included 44 children aged approximately 5 years. Participants were assigned to a Play group or a Control group. The Play group took part in 20 sessions of group role play with adult participation, organized around cultural texts and open-ended materials. Measures included children’s perceived stress, teacher-reported child stress, and internalizing and externalizing stress symptoms assessed through the Strengths and Difficulties Questionnaire. The results revealed that the Play group demonstrated lower perceived child stress and higher prosocial behavior compared to the Control group at post-test. Gain score analyses showed significant improvement differences in teacher-reported child stress, conduct problems, peer problems, and prosocial behavior between the groups. No significant effects were found for emotional symptoms, suggesting that the intervention had a stronger impact on externalizing than internalizing manifestations of stress. These findings indicate that group-based role play with adult participation may be an accessible tool for reducing externalizing manifestations of stress and supporting social functioning in preschool settings.

## 1 Introduction

One of the latest definitions describes stress as the general adaptive reaction to perceived or actual threats, which heightens both physiological and psychological functions to manage the stressor (McEwen and Akil, 2020). Modern children are exposed to an increasing number of stressful life events, including family-related difficulties (e.g., loss of close others, divorce, neglect, financial hardship), as well as broader socio-economic and environmental threats such as disasters, conflicts, and rapid societal changes (Petrash, 2025; Schwarzer and Schulz, 2003; UNDRR, 2020). Recent data indicate that up to 48.4% of families report stressful events in early childhood (Luo et al., 2023; Shaigerova et al., 2024). In addition, every day educational environments can also serve as a source of stress for young children. Studies show that cortisol levels in preschoolers tend to increase over the course of the day in kindergarten settings (de Vet et al., 2023; Gavrilova et al., 2025).

Stress in early childhood is typically expressed through both internalizing and externalizing symptoms. Internalizing symptoms include anxiety, withdrawal, and broader emotional difficulties, whereas externalizing symptoms manifest as aggression, impulsivity, and conduct problems (Perren et al., 2007; Poulou, 2015). These early symptoms can serve as predictors of long-term negative outcomes if not addressed, including poor cognitive function, lower academic achievement, and increased risk of mental health problems such as anxiety and depression (Fomina et al., 2025; Goodman et al., 2012; LeMoult et al., 2020). Given these risks, early identification and prevention of stress-related symptoms in preschool children is of critical importance. Therefore, there is a growing need to develop and evaluate intervention methods that can be effectively applied in preschool classrooms and delivered not only by psychologists but also by regular teachers.

### 1.1 The potential of role play for stress reduction in preschool children

One of the most effective approaches to the theoretical analysis of play as a phenomenon is considered a cultural-historical approach. Unlike many contemporary approaches that examine play primarily as a context associated with particular developmental outcomes—such as executive function (White et al., 2021), language development (Wasik and Jacobi-Vessels, 2017), social competence (Smits-van der Nat et al., 2024), or school readiness (Yogman et al., 2018)—the cultural-historical approach considers play within the context of its genesis and development. Rather than focusing solely on the functions or outcomes of play, it seeks to uncover the developmental mechanisms through which play contributes to child development. It not only has great theoretical potential but also offers an arsenal of tools for analyzing play as a phenomenon (Smirnova et al., 2010; Veraksa et al., 2025; Veresov and Veraksa, 2025). According to the cultural-historical approach, role play is the leading activity in preschool age, it serves as the primary context for the emergence of key psychological formations during this period (Elkonin, 2005). It involves the symbolic enactment of social relationships from the “adult world” within imaginary situations, ranging from simple play with toys to complex group-based dramatizations of narratives. Role play provides not only enjoyment but also substantial developmental benefits (Zaporozhets and Elkonin, 1965; Vygotsky, 2016). Role play contributes to the development of speech, self-regulation, and socio-emotional competence (Smits-van der Nat et al., 2024; Wasik and Jacobi-Vessels, 2017; White et al., 2021). Despite the developmental potential of role play, an important question remains: which features of play may contribute to stress reduction?

First, role play facilitates the reduction of emotional tension. Symbolic nature of play creates a safe relational space in which children can express and explore their inner world—feelings, thoughts, and experiences—through a natural form of activity (Landreth, 2012; Shishova and Khotinets, 2025). Within role play, children express personal meanings and emotional experiences (Solovieva and Quintanar, 2021; Veraksa, 2011). Through role play, children can externalize and process their emotions, experiences, and internal states, allowing them to cope with emotional difficulties. Within this safe space, children symbolically process and overcome stressful and potentially traumatic experiences. These processes may underlie the potential of role play to reduce children’s subjectively perceived stress and alleviate internalizing symptoms associated with stress, including anxiety, emotional tension, and emotional difficulties.

Second, role play serves as a key context for the development of self-regulation in preschool age, including emotional regulation (Vygotsky, 2016). Its core components are the imaginary situation and the roles adopted by children. An imaginary situation requires children to continuously coordinate and shift between real and imaginary levels. At the same time, roles introduce a system of rules that children are expected to follow. Importantly, adherence to these rules becomes more manageable within play, as the intrinsic motivation and enjoyment derived from play outweigh the tendency toward impulsive action (Vygotsky, 2016). For these reasons, role play is considered a primary context for the development of self-regulation in early childhood. Therefore, children become better able to regulate their behavior and emotions. Consequently, the self-regulatory potential of role play may contribute to reducing both internalizing and externalizing manifestations of stress.

Third, role play provides a context for practicing skills, including coping with stressful situations and challenging emotions. Imaginary situations allow children to enact different scenarios and experiment with possible actions and responses. This creates opportunities to refine behavioral strategies and develop more adaptive ways of responding to social and emotional challenges. Through repeated practice in a safe and imaginary context, children may acquire more effective coping strategies, which can contribute to reducing externalizing manifestations of stress, such as impulsive reactions and behavioral difficulties.

Fourth, role play provides a space for social interaction and connection with peers, which is a crucial resource for coping with stress. Within role play, children interact on two levels simultaneously: the real level (e.g., negotiating the plot, organizing materials) and the imaginary level (e.g., speaking in character). This dual interaction supports the development of social competence and strengthens a sense of connectedness with others. As social support and positive peer relationships are important protective factors against stress (Butler et al., 2022), these processes may contribute to reducing externalizing manifestations of stress, particularly peer conflicts and behavioral difficulties in social interactions.

Thus, role play represents a space in which children can express personal meanings and experiences in a safe environment, develop self-regulation and coping skills, and build social connections with peers and adults. Forms of role play are widely used in clinical and therapeutic practice to reduce symptoms of stress, anxiety, aggression, and PTSD in preschool children (Bratton et al., 2005; LeBlanc and Ritchie, 2001; Parker et al., 2021; Patterson, 2021). However, the application of role play as a therapeutic method requires extensive and specialized training (Landreth and Sweeney, 2013). In addition, therapeutic interventions are typically long-term: meta-analyses suggest that measurable effects of play therapy often emerge after approximately 30 sessions, corresponding to around 6 months of intervention (LeBlanc and Ritchie, 2001). Furthermore, role play and other forms of play in therapeutic contexts are most often used in individual formats (Davenport and Bourgeois, 2008; Hateli, 2022). Taken together, these factors limit the accessibility of play-based therapy for preschool children. At the same time, the symbolic nature of play, together with its broad impact on cognitive and socio-emotional development, suggests that group role play in kindergarten contexts may represent a promising and accessible approach for reducing stress, mitigating its negative effects, and fostering psychological resilience. However, this assumption requires empirical validation.

### 1.2 The role of an adult in role play

Within the cultural-historical approach, the adult is viewed as a bearer of cultural experience, meanings, and ideal forms of activity, guiding the child’s development through participation in shared activity (Dubrovina, 2013; Veresov et al., 2021). This principle also applies to role play. In role play, children reproduce and reinterpret situations from social life, thereby appropriating cultural meanings, roles, and forms of interaction (Elkonin, 2005; Shuttleworth, 2011). However, rich and mature role play requires a broad range of abilities that are often still within the child’s zone of proximal development, including the ability to construct and sustain an imaginary situation, develop a coherent plot, organize the play space, use substitute objects, coordinate actions with peers, and maintain role behavior over time (Smirnova, 2015; Sukhikh et al., 2025).

For this reason, adult participation may play an important developmental and therapeutic role in supporting children’s role play. The adult provides orientation within the play situation by introducing cultural content, modeling roles and actions, enriching the narrative structure of play, and supporting children’s understanding of the logic of the imaginary situation (Solovieva and Quintanar, 2019). Through such guidance, the adult helps children navigate both the symbolic and social dimensions of role play. Adult involvement may therefore expand the scale, complexity, and duration of role play and create conditions for higher levels of child participation and development (Fleer et al., 2017; Verenikina, 2003).

At the same time, the question of optimal adult involvement in role play remains open. Researchers emphasize the importance of maintaining a balance between support and child initiative (Whitebread and O’Sullivan, 2012). Excessive adult control may transform role play into dramatization or theatrical performance, reducing children’s agency and subjectivity within role play (McCabe, 2017). Therefore, the role of the adult is not to impose ready-made scenarios but to sensitively scaffold the development of role play while preserving its voluntary, imaginative, and child-directed nature.

### 1.3 Current study

Thus, the present study aimed to examine the potential of group role play in a kindergarten setting as a means of reducing stress and its manifestations in preschool children.

Given that more mature forms of role play are associated with stronger developmental outcomes, it is essential to create conditions that support rich and elaborated play (Sukhikh et al., 2025). An adult, as a carrier of cultural knowledge and experience, can enrich children’s play by introducing new roles, actions, and narrative structures (Veresov and Veraksa, 2025). In addition, relying on the narrative structure of a cultural text as a framework can further enrich role play (Iakshina and Smirnova, 2024; Veresov and Veraksa, 2025). For these reasons, the present study incorporated both a cultural text and an adult as a play partner into the group role play intervention.

Based on the cultural-historical approach to play and child development (Elkonin, 2005; Zaporozhets and Elkonin, 1965; Veresov, 2024; Vygotsky, 2016), the following hypotheses were proposed:

1. Group role play with adult participation would be associated with a reduction in children’s subjectively perceived stress.
2. Group role play with adult participation would be associated with a reduction in internalizing symptoms of stress.
3. Group role play with adult participation would be associated with a reduction in externalizing symptoms of stress.

## 2 Methods

### 2.1 Participants

The initial sample consisted of 50 preschool children (Mean age = 63.2 months, SD = 4.7 months) with a high level of perceived stress, as measured by the Perceived Stress Scale for Children (PSS-C). The final sample included 44 children (Mean age = 63.6 months, SD = 4.65 months, 14 boys and 30 girls). Six children were excluded from the analysis: five due to attending fewer than half of the intervention sessions and one due to absence at the post-test assessment.

All participants attended one of four public kindergartens in Moscow located in districts characterized by a moderate level of infrastructure and predominantly serving middle-class families. Written informed consent was obtained from the parents of all participants. The sample size was determined by the number of kindergartens and teachers who allowed the intervention. Not all children from the classes participated in the intervention, some just followed the usual curriculum. This allowed the formation of two groups equal in sex, age, and initial level of perceived stress. Teachers were not informed about the specific aims and hypotheses of the intervention study.

The study was approved by the Ethics Committee of the Federal Scientific Center of Psychological and Multidisciplinary Research (31.01.2024, No. 2).

### 2.2 Procedure

The study employed an experimental design with two conditions (Play group vs. Control group) and repeated measures (pre-test and post-test). At the initial stage, 210 preschool children were assessed for perceived stress using the PSS-C and executive functions (EF, Sidneva, 2025) using NEPSY-II subtests for working memory and inhibition (Korkman et al., 2007; Veraksa et al., 2020), and test “Dimensional change card sort” (Zelazo, 2006; Almazova et al., 2024) for cognitive flexibility. Children were classified into three levels of EF development (low, medium, and high) based on the results of a *K*-means cluster analysis of EF assessment scores. EF were included as a control variable in the study to allow for a more accurate estimation of the intervention effects.

Based on the obtained perceived stress scores, children in the upper quartile (scores ≥ 16 out of 24) were selected for participation in the study. A pre-test assessment was then conducted with the selected participants. It included teacher-reported measures of child stress and children’s socio-emotional functioning, including both externalizing and internalizing symptoms. Following the pre-test, children were randomly assigned to the Play (*N* = 25) and Control groups (*N* = 25). The groups were matched to ensure equivalence in terms of sex, age, and baseline stress and EF levels.

The intervention consisted of 20 sessions lasting 25–30 min each. The sessions were conducted in kindergarten settings, in quiet and well-lit rooms familiar to the children. Activities were carried out in mini-groups of 5–6 children. The intervention was delivered by two specially trained psychologist-experimenters under ongoing supervision to reduce the risk of experimenter effects. The same experimenters worked with the children throughout the intervention period, ensuring consistency of interaction and minimizing potential adaptation-related stress. Sessions 1–2, 9–10, and 17–18 in each group were video-recorded and reviewed to evaluate treatment fidelity, monitor the quality of implementation, and ensure compliance with the standardized intervention protocol.

Following the completion of the intervention, a post-test assessment was conducted that included measures of perceived stress (PSS-C), teacher-reported measures of child stress and children’s socio-emotional functioning, including both externalizing and internalizing symptoms. After the post-test assessment, the final sample consisted of 25 children in the Сontrol group and 19 children in the Play group.

### 2.3 Measures

The Russian version of the Perceived Stress Scale for Children (PSS-C) was used to assess children’s self-reported stress (Cohen et al., 1983; Kornienko et al., 2024). The scale consists of 10 items rated on a 4-point scale (from 1 “never” to 4 “almost always”) and includes two subscales: Stress (Distress, max. 24 scores) and Well-being (max. 16 scores). The Distress Scale includes six items that assess different dimensions of stress that the child experienced last week (rushing or hurrying, feeling upset or angry, etc.). The Scale of Well-Being includes four items that assess different positive experiences they faced over the last week (feeling happy, parent support and love, etc.).

The Russian version of the Perceived Child Stress Scale (teacher version) was used to assess children’s stress as reported by adults (Cohen et al., 1983; Kornienko et al., 2024). The questionnaire includes 6 items rated on a 4-point scale (from 1 “never” to 4 “almost always”), reflecting behavioral and emotional manifestations of stress during the past week.

The Russian version of the Strengths and Difficulties Questionnaire (SDQ) (Goodman, 2001; Goodman et al., 2005) was used to assess children’s socio-emotional functioning based on teacher reports. The questionnaire includes 25 items rated on a 5-point scale (from 1 “not true” to 5 “certainly true”) and comprises five subscales: Emotional Symptoms, Conduct Problems, Hyperactivity/Inattention, Peer Problems, and Prosocial Behavior. Together, these subscales capture the most common internalizing and externalizing manifestations of stress in preschool children.

### 2.4 Intervention

The intervention in Play group was based on the “Playworlds” approach (Fleer, 2021; Lindqvist, 1996). Each cycle of sessions was organized around a narrative framework derived from children’s literature: Ludwig the Fourteenth and Tutta Karlsson by Jan Ekholm (1–10 sessions) and The Incredible Story of the Giant Pear by Jakob Martin Strid (11–20 sessions). The books were selected to ensure novelty for the children, thereby sustaining engagement and motivation. The stories served as a basis for role distribution, development of play scenarios, and emotional engagement. Interactive reading techniques were used to introduce the story, followed by role play with an adult acting as a play partner. Strict adherence to the original storyline during role play was not required, allowing children to introduce their own interpretations and modifications of the narrative. The narratives emphasized themes of overcoming challenges through social support (friends and family), developing self-efficacy and self-regulation, mobilizing internal resources, and confronting fear.

The main character of Ludwig the Fourteenth and Tutta Karlsson is Ludwig the Fourteenth, a young fox born into a fox family where cunning, deception, and hunting chickens are considered normal. However, Ludwig is very different from the other foxes: he is honest, kind, and unwilling to harm others. One day, he meets Tutta Karlsson, a chick from a chicken family. Although foxes and chickens are traditionally regarded as enemies, the two develop a friendship. Together, the characters encounter various dangers (such as fox hunts, the risk of their friendship being discovered, and situations requiring rescue), learn to trust one another, and help their families overcome fear and hostility. Accordingly, during sessions 1–10, the main play themes included: family relationships and everyday family life, friendship-based adventures (e.g., rescuing a fox cub or a chick, surviving a storm in the forest), animal school days, social conflicts and their resolution, visiting one another, and supporting friends and siblings in interactions with parents. In the role play based on Ludwig the Fourteenth and Tutta Karlsson, children most frequently enacted family conflicts (e.g., between parents and children or between siblings) and collaboratively explored different ways of resolving them through play.

The Incredible Story of the Giant Pear tells the story of two friends — Sebastian the elephant and Mitcho the cat. One day, they discover a bottle containing a message from the missing mayor of their town, who has become stranded on a mysterious island. Along with the message, the characters also discover an unusual giant pear. Sebastian and Mitcho transform the enormous pear into a boat and embark on a dangerous sea journey to rescue the mayor. Along the way, they encounter various challenges and adventures, including storms, pirates, sea creatures, and unknown islands. Throughout the journey, the characters support one another, overcome fear, and demonstrate courage, resourcefulness, and friendship. Accordingly, the main theme of sessions 11–20 included different adventures (e.g., building a ship, sailing across the sea, treasure hunting, exploring new islands, meeting new characters on the islands, and overcoming mountains and chasms), as well as organizing celebrations after overcoming fears or at the end of the journey. In the play scenarios based on The Incredible Story of the Giant Pear, children most frequently enacted situations involving helping behavior, in which many friends came together to support one another, as well as situations requiring courage and bravery in the face of danger or uncertainty.

The play environment was organized using flexible, open-ended materials (e.g., fabrics, paper, ribbons), allowing children to construct costumes and play attributes. Character costumes were created using simple symbolic elements, including hats (e.g., yellow hats for chickens and chicks), ribbons (e.g., orange ribbons tied around the waist and used as fox tails), and pieces of fabric (e.g., black fabric used as pirate capes). Fabrics, chairs, and soft poufs were also used to create play locations and imaginary settings, such as the fox family’s house or a ship during sea adventures. In addition, the play environment included various objects that were not directly intended as costumes or locations but could be used by children as symbolic substitute objects during play (e.g., cardboard boxes, plastic cups, pinecones, stones, and other open-ended materials).

Each session included the following stages: (1) interactive reading and discussion of the story, (2) role assignment, (3) a transition ritual marking the entry into the imaginary play world, (4) putting on the costumes made from open materials, (5) group role play with the adult, and (6) a transition ritual marking the return from the imaginary play world to reality, followed by a guided reflection and discussion of the play experience. The adult maintained a role within the play, supporting children’s engagement, sustaining the imaginary situation, and encouraging children’s initiative and modifications of the plot.

The Control group did not receive any intervention and followed the regular kindergarten routine.

Both groups attended kindergarten from 9:00 a.m. to 5:00 p.m. They followed the standard Russian preschool education curriculum, based on the “From Birth to School” program developed under the editorship of Veraksa et al. (2019). Standard activities within the curriculum included productive activities (e.g., drawing, modeling, and crafts), mathematics classes, physical education, dance and music activities, reading sessions, outdoor activities, and activities focused on learning about the surrounding world, nature and culture.

### 2.5 Data analysis

Chi-square tests were conducted to examine group equivalence in categorical variables (e.g., sex distribution). Group differences at pre-test and post-test were examined using the Kruskal–Wallis test (one-way ANOVA on ranks). Gain scores (post-test minus pre-test) were calculated to assess changes over time, and group differences in gain scores were also examined using the Kruskal–Wallis test (one-way ANOVA on ranks). Statistical significance was set at *p* < 0.05 for all analyses. Effect sizes for group differences in gain scores were estimated using epsilon-squared (*ε*^2^). Given the small sample size, non-parametric tests were used.

## 3 Results

### 3.1 Preliminary analysis and descriptive statistics

A one-way ANOVA on ranks (Kruskal–Wallis test) revealed no significant differences between the Play and Control groups at pre-test across all measured variables (*p* > 0.05 for all comparisons), indicating baseline equivalence between groups. Descriptive statistics for all study variables at pre-test are presented in Table 1, for study variables at post-test – in Table 2. Significant differences between boys and girls were found for Perceived Child Stress (*χ*^2^ = 4.6603, *p* = 0.031), Child Stress (teacher-reported) (*χ*^2^ = 5.4333, *p* = 0.020), Hyperactivity (*χ*^2^ = 15.3231, *p* < 0.001), Conduct Problems (*χ*^2^ = 10.5407, *p* = 0.001), and Emotional Symptoms (*χ*^2^ = 8.3640, *p* = 0.004). Importantly, the Play and Control groups did not differ in sex composition (*N* = 44, *χ*^2^ = 0.0008, *p* = 0.976), indicating that the groups were comparable in terms of sex distribution. Therefore, subsequent analyses of group differences were conducted without additional controls for sex.

**Table 1:** Pre-test descriptive statistics.

Parameters	Play group		Control group		Kruskal–Wallis test
	Mean	Standard deviation	Mean	Standard deviation	
Perceived child stress	17.68	1.6	18.68	2.17	*χ*^2^ = 2.1731, *p* = 0.140, *ε*^2^ = 0.04
Perceived child well-being	13.0	2.45	13.52	1.92	*χ*^2^ = 0.3885, *p* = 0.533, *ε*^2^ = 0.009
Child stress (teacher-reported)	14.0	3.53	11.96	3.38	*χ*^2^ = 3.0663, *p* = 0.080, *ε*^2^ = 0.07
Hyperactivity/Inattention	13.68	4.28	13.88	3.73	*χ*^2^ = 0.0410, *p* = 0.840, *ε*^2^ = 0.009
Conduct problems	11.05	4.95	10.72	3.66	*χ*^2^ = 0.0627, *p* = 0.802, *ε*^2^ = 0.001
Peer problems	11.95	3.49	10.28	2.73	*χ*^2^ = 2.9534, *p* = 0.086, *ε*^2^ = 0.06
Prosocial behavior	16.37	3.53	18.12	3.48	*χ*^2^ = 2.1625, *p* = 0.141, *ε*^2^ = 0.05
Emotional symptoms	13.16	3.72	11.68	3.39	*χ*^2^ = 2.5545, *p* = 0.110, *ε*^2^ = 0.05

Scores are expressed in raw scale points.

**Table 2:** Post-test descriptive statistics.

Variables	Play group		Control group		Kruskal–Wallis test
	Mean	Standard deviation	Mean	Standard deviation	
Perceived child stress	14.32	2.36	16.64	3.87	***χ***^***2***^ **= 4.061, *p* = 0.044, *ε***^**2**^ **= 0.09**
Perceived child well-being	13.11	2.64	12.8	2.68	*χ*^2^ = 0.176, *p* = 0.675, *ε*^2^ = 0.004
Child stress (teacher-reported)	13.21	3.49	13.8	3.86	*χ*^2^ = 0.356, *p* = 0.551, *ε*^2^ = 0.008
Hyperactivity/inattention	13.05	4.42	14.28	5.6	*χ*^2^ = 0.353, *p* = 0.552, *ε*^2^ = 0.008
Conduct problems	10.32	4.77	12.12	5.68	*χ*^2^ = 1.471, *p* = 0.225, *ε*^2^ = 0.03
Peer problems	11.0	3.43	12.52	3.14	*χ*^2^ = 2.140, *p* = 0.143, *ε*^2^ = 0.04
Prosocial behavior	17.95	3.7	15.04	4.5	***χ***^**2**^ **= 5.264, *p* = 0.022, *ε***^**2**^ **= 0.122**
Emotional symptoms	12.47	3.88	11.48	5.64	*χ*^2^ = 1.420, *p* = 0.233, *ε*^2^ = 0.03

Scores are expressed in raw scale points. Statistically significant differences are shown in bold.

### 3.2 Group comparisons

To ensure the robustness of the findings, group differences were examined both at the post-test stage and in gain scores (i.e., changes from pre-test to post-test).

At post-test (Table 2), the Kruskal–Wallis test revealed significant differences between the Play and Control groups for Perceived Child Stress (*χ*^2^ = 4.061, *p* = 0.044, *ε*^2^ = 0.09) and Prosocial Behavior (*χ*^2^ = 5.264, *p* = 0.022, *ε*^2^ = 0.122). In all these cases, the Play group demonstrated more favorable outcomes, including lower levels of perceived child stress and teacher stress, as well as higher levels of prosocial behavior.

Significant group differences in gain scores were observed for Child Stress (teacher-reported) (*χ*^2^ = 4.698, *p* = 0.030, *ε*^2^ = 0.109), Conduct Problems (*χ*^2^ = 4.588, *p* = 0.032, *ε*^2^ = 0.107), Peer Problems (*χ*^2^ = 10.526, *p* = 0.001, *ε*^2^ = 0.245), and Prosocial Behavior (*χ*^2^ = 8.413, *p* = 0.004, *ε*^2^ = 0.196). The Play group showed greater improvements over time, including reductions in stress and behavioral difficulties, as well as an increase in prosocial behavior. No other significant group differences were observed. Descriptive statistics and the results of the gain score analysis are presented in Table 3.

**Table 3:** Group differences in gain scores: descriptive statistics and Kruskal–Wallis test results.

Gain in variable	Play *M* (SD)	Control *M* (SD)	*χ* ^2^	*p*	Effect size (*ε*^2^)
Gain in perceived child stress	−3.368 (2.629)	−2.040 (3.691)	0.875	0.349	0.02036
Gain in perceived child well-being	0.105 (3.230)	−0.720 (3.458)	0.314	0.575	0.00730
Gain in child stress (teacher-reported)	−0.789 (1.584)	1.840 (4.767)	4.698	**0.030**	0.10926
Gain in hyperactivity	−0.632 (2.033)	0.400 (3.830)	0.832	0.362	0.01934
Gain in conduct problems	−0.737 (1.628)	1.400 (3.428)	4.588	**0.032**	0.10670
Gain in peer problems	−0.947 (2.460)	2.240 (3.205)	10.526	**0.001**	0.24480
Gain in prosocial behavior	1.579 (2.854)	−3.080 (5.016)	8.413	**0.004**	0.19565
Gain in emotional symptoms	−0.684 (1.668)	−0.200 (4.610)	0.104	0.747	0.00243

Statistically significant differences are shown in bold.

## 4 Discussion

### 4.1 Role play effects on stress reduction in preschool children

The present study aimed to examine the potential of group-based role play in a kindergarten setting to reduce stress and its manifestations in preschool children. Overall, the findings provide partial support for the proposed hypotheses. First, group role play with adult participation was associated with a reduction in children’s subjectively perceived stress at post-test; however, this effect was not confirmed in the gain score analysis, indicating only partial support for the first hypothesis. Second, the hypothesis regarding internalizing symptoms was not supported, as no significant differences were found for emotional symptoms. Third, the findings support the hypothesis regarding externalizing manifestations of stress. Significant improvements were observed in teacher-reported child stress and reductions in conduct and peer problems, as well as an increase in prosocial behavior in the Play group compared to the Control group.

Why did group-based role play contribute to a reduction in externalizing symptoms of stress? From the perspective of cultural-historical psychology, roles in play inherently introduce a system of rules that must be followed by the players (Elkonin, 2005; Vygotsky, 2016). Importantly, these rules are not externally imposed but are internally accepted through engagement in the role, play, and joint activity, which makes them easier to follow. Due to the strong affective involvement in play, children are better able to inhibit impulsive reactions and act in accordance with the logic of the role (Vygotsky, 2016). Moreover, in role play, children monitor not only their own behavior but also that of their peers, and this shared regulation of rule-following supports better regulation of impulsive responses (Veresov and Veraksa, 2025). In this way, role play supports the development of voluntary regulation, as children learn to control their behavior and mental processes. These changes become visible in everyday classroom behavior and are reflected in teacher reports, which may explain the observed reduction in conduct problems and other externalizing manifestations of stress.

In addition, the presence of an adult as a play partner contributed to the enrichment of play and the increase in its level. Acting as a model of mature play behavior, the adult introduced more complex roles, actions, and interactions, thereby supporting children in reaching higher levels of play (Sukhikh et al., 2025). As children become more competent and successful participants in this leading activity, they are more readily included in peer interactions, which may reduce stress through increased social support within joint activity (Hostinar and Gunnar, 2015; Veraksa et al., 2024). Furthermore, role play shifts the focus from competition to cooperation, as children are engaged in a common narrative and shared goals rather than individual achievement (Solovieva and Quintanar, 2021). Finally, the dual structure of play – simultaneously operating on real and imaginary levels – requires children to negotiate, coordinate actions, and take others’ perspectives (Veraksa, 2022). These foster cooperation and mutual regulation, which in turn may explain the reduction in peer problems observed in the Play group.

Why did group-based role play have a weaker effect on internalizing symptoms of stress? First, internalizing symptoms are closely related to children’s personal experiences and may require more individualized support. The reduction of internalizing symptoms often depends on processes of emotional verbalization and reflection (Halfon et al., 2017). Although such elements were partially presented in the intervention, in group-based role play opportunities for expressing personal meanings and emotional experiences may be limited, as children need to coordinate their actions within a shared narrative. Moreover, the use of predefined cultural texts as a basis for play, while enriching the structure of the activity, may not always resonate with each child’s personal emotional experiences. Third, the duration of the intervention may have been insufficient to produce observable reductions in anxiety and emotional distress. Although the program included 20 sessions, internalizing symptoms are often relatively stable and may require longer or more intensive interventions to change. Finally, internalizing symptoms, such as anxiety and emotional distress, are less overt and therefore less observable in everyday behavior, particularly from the perspective of teachers. As a result, changes in these symptoms may be more difficult to detect using teacher-reported measures.

The absence of significant changes in perceived well-being may reflect the fact that well-being is not simply the opposite of perceived stress, but a distinct psychological construct. Although reductions in stress may contribute to improved well-being, psychological well-being is also associated with more stable factors, including a child’s sense of security, trust in significant others, and the availability of supportive attachment relationships (Bowlby, 1988; Sroufe, 2005). As a result, well-being may be less sensitive to short-term changes than stress-related symptoms and may require broader or longer-lasting changes in a child’s environment to show measurable improvement. Therefore, a 20-session intervention may have been sufficient to reduce certain manifestations of stress without producing measurable changes in children’s overall well-being.

### 4.2 Limitations

Several limitations of the present study should be acknowledged. First, the relatively small final sample size (*N* = 44) limits the statistical power and generalizability of the findings. At the same time, this limitation is partially mitigated by the use of complementary statistical approaches (e.g., analysis of both post-test differences and gain scores), as well as multiple measures, which together provide a more robust and comprehensive assessment of stress and its manifestations. Second, several outcomes were based on teacher reports. Internalizing symptoms are less observable in everyday classroom interactions, and teacher-reported measures may be insufficiently sensitive to subtle changes in anxiety and emotional distress. Third, the Control group did not participate in an alternative activity of comparable intensity. Therefore, some of the observed effects may be partially attributed to increased adult attention and engagement in structured group activity, rather than to role play *per se*.

### 4.3 Practical implications

1. Group-based role play can be used as an accessible tool for reducing behavioral manifestations of stress in preschool settings. The findings suggest that role play with adult participation may be effectively integrated into everyday kindergarten practice to reduce externalizing symptoms, such as conduct problems and peer difficulties, and perceived child stress without requiring individualized therapeutic intervention.
2. Adult involvement is a key condition for enhancing the developmental and regulatory potential of role play. The presence of an adult as a play partner supports the enrichment of play, promotes higher levels of play development, and facilitates the emergence of self-regulation and cooperative behavior. Therefore, training educators to participate in role play in a developmentally appropriate way may increase the effectiveness of such interventions.
3. Additional components may be needed to address internalizing symptoms of stress. Given the limited effects of group role play on internalizing symptoms, interventions may benefit from incorporating elements that support emotional verbalization and reflection, as well as opportunities for more individualized expression within group activities.

The findings may be applied in preschool practice, particularly in the design of group-based interventions aimed at improving classroom climate, enhancing self-regulation, and preventing stress-related behavioral difficulties.

### 4.4 Future directions

Future research should further explore the mechanisms underlying the effects of role play, including the role of emotional reflection and individual expression, as well as examine the long-term impact of such interventions and their effectiveness when implemented by regular educators in naturalistic classroom settings.

## Data Availability

The original contributions presented in the study are included in the article/supplementary material, further inquiries can be directed to the corresponding author.
